# Supplementary material for: Empirical methods for controlling false positives and estimating confidence in ChIP-Seq peaks
Source: BMC Bioinformatics. 2008 Dec 5;9:523. doi: 10.1186/1471-2105-9-523 (PMC2628906; doi:10.1186/1471-2105-9-523)
Supplement: Additional File 1 — A variety of html documents from the USeq web site detailing the available applications, their best usage, output file type descriptions, command line menus, etc. [file 1471-2105-9-523-S1.zip › USeqUserGuides/JavaDoc.zip!/JavaDoc/bioroot/class-use/Preference.html]

Uses of Class bioroot.Preference


|  |  |  |
| --- | --- | --- |
| **Overview** \| **Package** \| **Class** \| **Use** \| **Tree** \| **Deprecated** \| **Index** \| **Help** | |  |
| PREV   NEXT | **FRAMES**    **NO FRAMES**     **All Classes** |  |


---


## **Uses of Class bioroot.Preference**

| Packages that use Preference | |
| --- | --- |
| **bioroot** |  |
| **bioroot.oligo** |  |
| **bioroot.strain** |  |

Uses of Preference in bioroot

| Methods in bioroot that return Preference | |
| --- | --- |
| `Preference` | `UserBean.getAntibodyPreference()` |
| `Preference` | `UserBean.getOligoPreference()` |
| `Preference` | `UserBean.getPlasmidPreference()` |

| Methods in bioroot with parameters of type Preference | |
| --- | --- |
| `static java.lang.String` | `Util.fetchTableColumnRow(Preference pref, java.lang.String reagentType)`             Returns a table header row using the Preferece obj and a reagentType: Oligo, Plasmid, Strain... |
| `static java.lang.String` | `Util.getViewLine(ReagentBean[] reagentBeans, int[] inView, Preference reagentPreference, java.lang.String reagentType)`             Returns hot linked html for 1-30 31-60 ... |
| `void` | `UserBean.setAntibodyPreference(Preference antibodyPreference)` |
| `void` | `UserBean.setOligoPreference(Preference oligoPreference)` |
| `void` | `UserBean.setPlasmidPreference(Preference plasmidPreference)` |

Uses of Preference in bioroot.oligo

| Methods in bioroot.oligo with parameters of type Preference | |
| --- | --- |
| `static java.lang.String` | `OligoSpreadSheet.fetchOligoTableRows(Preference pref, OligoBean[] beans, int[] startStop, DBUtil bioRoot, boolean checked, boolean allTabs)`             Returns table rows for every oligo according to an OligoPreference, if allTabs==true, then all features are returned in a tab delimited format, otherwise its HTML. |
| `static OligoBean[]` | `OligoSpreadSheet.orderOligos(java.lang.String featureName, Preference pref, OligoBean[] beans, DBUtil bioRoot)`             Orders oligos, sort of nasty, can't figure another way, reflection is way too slow. |

Uses of Preference in bioroot.strain

| Subclasses of Preference in bioroot.strain | |
| --- | --- |
| `class` | `StrainPreference` |

---


|  |  |  |
| --- | --- | --- |
| **Overview** \| **Package** \| **Class** \| **Use** \| **Tree** \| **Deprecated** \| **Index** \| **Help** | |  |
| PREV   NEXT | **FRAMES**    **NO FRAMES**     **All Classes** |  |


---
